# Modeling Non-Alcoholic Fatty Liver Disease (NAFLD) Using “Good-Fit” Genome-Editing Tools

**DOI:** 10.3390/cells9122572

**Published:** 2020-12-01

**Authors:** Uijin Kim, Nahyun Kim, Ha Youn Shin

**Affiliations:** Department of Biomedical Science & Engineering, Konkuk University, Seoul 05029, Korea; rladmlwls135@naver.com (U.K.); knh64@naver.com (N.K.)

**Keywords:** NAFLD, genome engineering, CRISPR/Cas9, base editor, prime editor

## Abstract

Non-alcoholic fatty liver disease (NAFLD), which affects both adults and children, is the most common liver disorder worldwide. NAFLD is characterized by excess fat accumulation in the liver in the absence of significant alcohol use. NAFLD is strongly associated with obesity, insulin resistance, metabolic syndrome, as well as specific genetic polymorphisms. Severe NAFLD cases can further progress to cirrhosis, hepatocellular carcinoma (HCC), or cardiovascular complications. Here, we describe the pathophysiological features and critical genetic variants associated with NAFLD. Recent advances in genome-engineering technology have provided a new opportunity to generate in vitro and in vivo models that reflect the genetic abnormalities of NAFLD. We review the currently developed NAFLD models generated using clustered regularly interspaced short palindromic repeats/CRISPR-associated protein 9 (CRISPR/Cas9) genome editing. We further discuss unique features of CRISPR/Cas9 and Cas9 variants, including base editors and prime editor, that are useful for replicating genetic features specific to NAFLD. We also compare advantages and limitations of currently available methods for delivering genome-editing tools necessary for optimal genome editing. This review should provide helpful guidance for selecting “good fit” genome-editing tools and appropriate gene-delivery methods for the successful development of NAFLD models and clinical therapeutics.

## 1. Introduction

### 1.1. Pathophysiology of Non-Alcoholic Fatty Liver Disease

**Figure 1:**
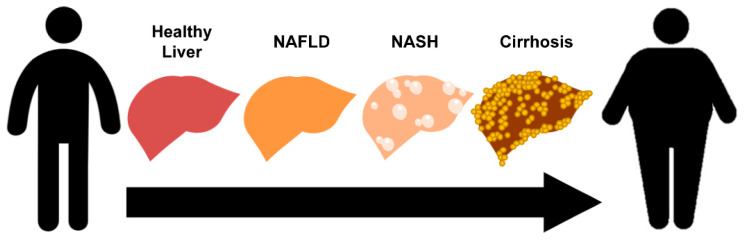
Schematic illustration of non-alcoholic fatty liver disease (NAFLD) progression.

Non-alcoholic fatty liver disease (NAFLD) is the leading cause of chronic liver disease with a worldwide prevalence of approximately 25% [1]. NAFLD is characterized by excess fat accumulation, primarily in the form of triglycerides (TGs), in more than 5% of the liver without a clear cause, such as excessive alcohol consumption (>20 g/d for women and >30 g/d for men), drug use, or viral hepatitis [2]. A subset of NAFLD patients progresses to non-alcoholic steatohepatitis (NASH) through hepatic injury, Mallory–Denk body (MDB) formation, inflammation or fibrosis (Figure 1) [3,4,5], and can further progress to cirrhosis, liver cancer, or cardiovascular complications [1,6,7,8]. The major risk factors for NAFLD are obesity, insulin resistance, and metabolic syndrome—collectively considered a pre-type 2 diabetes condition. Genetic polymorphisms are another important leading cause of NAFLD and may also be correlated with overweight status or metabolic disorder [9]. Genetic factors play critical roles in determining NAFLD occurrence, severity, and long-term prognosis. NAFLD can be diagnosed through blood tests by measuring serum levels of the liver injury markers, alanine aminotransferase (ALT) and aspartate aminotransferase (AST) [10]. Liver imaging using ultrasound or computed tomography (CT) scans can detect steatosis and cirrhosis, respectively. A liver biopsy is commonly used to examine the severity of inflammation and hepatic fibrosis. However, to date, there is no effective treatment for NAFLD other than weight loss through dietary change and exercise.

### 1.2. Genetic Variants Associated with NAFLD

Recent genome-wide association studies (GWAS) with single nucleotide polymorphism (SNP) analysis and independent cohort studies have revealed several key genetic variants associated with NAFLD (Table 1). Studying genetic factors that lead to NAFLD holds the key to a better understanding of the molecular pathogenesis underlying NAFLD occurrence and progression. Representative genetic variants associated with NAFLD are described below.

#### 1.2.1. PNPLA3

Patatin-like phospholipase domain-containing protein 3 (PNPLA3), also called adiponutrin, is a triglyceride (TG) lipase that mediates TG hydrolysis in fat cells. *PNPLA3* encodes a 481-amino acid protein that is abundantly expressed in human liver and mouse adipose tissue [9]. GWAS of three ethnic populations—Hispanic, African American, and European American—found a nonsynonymous SNP in *PNPLA3* (rs738409, C > G) that is highly associated with NAFLD [11]. This single variant results in substitution of guanine (G) for cytosine (C), changing an isoleucine to methionine at codon 148 (I148M) of PNPLA3. This specific SNP is strongly associated with increased levels of hepatic fats (>2-fold) in all three populations. Elevation of serum ALT and AST levels is more prevalent in the Hispanic population. Independent cohort studies further demonstrated that the *PNPLA3* rs738409 variant confers increased risk of NASH, fibrosis, and hepatocellular carcinoma (HCC) [12,13].

#### 1.2.2. TM6SF2

Transmembrane 6 superfamily 2 *(TM6SF2)* encodes a 351-amino acid protein that resides in the endoplasmic reticulum (ER) and the ER-Golgi intermediate compartment of human hepatocytes [9]. Although the biological function of *TM6SF2* has not been fully elucidated, small hairpin RNA (shRNA)-mediated knockdown of *TM6SF2* in mice impairs hepatocyte secretion of very low-density lipoprotein (VLDL) [14]. An exome-wide association study identified a specific SNP in *TM6SF2* (rs58542926, C > T, E167K) that is significantly associated with NAFLD. This single mutation is predominantly found in individuals of European ancestry. The *TM6SF2* rs58542926 variant is highly associated with increased levels of hepatic TGs and serum ALT. Additional case-control studies confirmed that the *TM6SF2* rs58542926 variant is strongly correlated with the severity of NASH, fibrosis, and HCC [15,16]. An expression quantitative trait locus (eQTL) analysis identified another *TM6SF2* variant (rs10401969, C > T) that is associated with reduced expression of *TM6SF2*. In vitro siRNA studies revealed that knockdown of TM6SF2 increased TG levels and lipid droplet area, whereas overexpression of TM6SF2 reduced lipid droplet content. These data suggest that TM6SF2 plays a critical role in TG metabolism and provide indirect evidence of an association of the *TM6SF2* variant (rs10401969) with NAFLD.

#### 1.2.3. GCKR

Glucokinase regulator (GCKR) is primarily expressed in the liver and plays a key role in controlling glucose metabolism by binding and transporting glucokinase [2]. A recent meta-analysis identified specific SNPs in *GCKR* (rs1260326 and rs780094) that are closely related to histological features of NASH [18]. These *GCKR* variants are associated with increased levels of TG and serum low-density lipoprotein (LDL)-cholesterol, but lower fasting glucose. A separate cohort study further verified the correlation of the *GCKR* rs780094 (C > T) variant with high levels of TG and serum glucose as well as with the severity of liver fibrosis [19].

#### 1.2.4. Other Genetic Variants that Influence NAFLD Susceptibility

Membrane-bound O-acyltransferase domain-containing 7 *(MBOAT7)* is highly expressed in hepatocytes and encodes an enzyme involved in reacylation of phospholipids in the context of phospholipid remodeling [9]. GWAS identified a specific SNP in *MBOAT7* (rs641738, C > T) that is associated with increased hepatic TG levels and NAFLD severity in patients of European descent [20,21]. Another study also demonstrated that children harboring the *MBOAT7* rs641738 variant show increased serum ALT levels [22]. GWAS of an Italian NAFLD cohort revealed that this single polymorphism increases the risk of HCC [23].

Heme oxygenase (HMOX1) is a key enzyme that protects the liver from oxidative stress caused by excessive heme [9]. Pediatric NAFLD patients who have genetic polymorphisms in the promoter region of *HMOX1* show increased serum ALT levels [26]. On the other hand, overexpression of HMOX1 in mice reduces hepatic TG levels and suppresses inflammatory responses and steatosis [24,25].

## 2. NAFLD Models Generated Using Genome Editing

Recent advances in genome-editing technology have provided unprecedented opportunities for establishing various disease models for biomedical research. Starting from zinc finger nucleases (ZFNs), researchers have developed transcription activator-like effector nucleases (TALENs) and clustered regularly interspaced short palindromic repeats/CRISPR-associated protein 9 (CRISPR/Cas9) genome-editing techniques [27]. To date, CRISPR/Cas9 has been the most widely used genome editing because of its relative ease of use as well as its time- and cost-effectiveness. In this review, we mainly discuss CRISPR/Cas9-mediated NAFLD models [28].

### 2.1. CRISPR/Cas9 Genome Editing

CRISPR/Cas9 was originally identified as the molecular basis of a prokaryotic immune system that confers protection against foreign pathogenic DNA [29]. Doudna and Charpentier re-engineered CRISPR/Cas9 as a more manageable two component system: a guide RNA (gRNA) and a Cas9 endonuclease [30]. Zhang and Church groups further applied this system for gene editing of cultured human cells [31,32]. Since then, CRISPR/Cas9 has been widely used for genome engineering of various organisms [33]. CRISPR/Cas9-mediated genome editing is a multi-step process that starts with gRNA recognition and complementary binding to a target genome sequence. Thereafter, Cas9 nuclease generates a double-strand break (DSB) three nucleotides upstream of a protospacer adjacent motif (PAM) with the sequence 5′-NGG-3′ (where “N” is any nucleotide) within the target region. Finally, a DNA repair system, either non-homologous end joining (NHEJ) or homology-directed repair (HDR), re-joins the DSB. The NHEJ-mediated repair system primarily generates deletion mutations, whereas HDR enables incorporation of a donor DNA template into the break point. Hence, CRISPR/Cas9 is commonly used to generate short deletions and does so with a relatively high deletion efficiency (up to 80%). Subsequent studies revealed that singly injecting gRNA into cells usually generates deletions less than 10 base pairs (bp) in length, whereas simultaneous injection of several gRNAs targeting multiple sites can generate large deletions up to several kilobases [34]. For replacement or insertion of specific sequences, a donor DNA template and CRISPR/Cas9 need to be simultaneously introduced into cells, although the insertion efficiency is dependent on cell type and target sequence.

### 2.2. CRISPR/Cas9-Mediated NAFLD Models

Although recent GWAS have identified genetic variants that are significantly associated with NAFLD, causal links between specific SNPs and NAFLD must be verified by functional analyses. Advances in genome-editing tools, particularly CRISPR/Cas9, have enabled the development of several in vitro and in vivo NAFLD models [28]. Representative CRISPR/Cas9-mediated NAFLD models are described below (Table 2).

#### 2.2.1. PNPLA3-Targeted Models

The *PNPLA3* rs738409 (I148M) variant is one of the best-characterized NAFLD-associated SNPs. To determine the biological function of this specific SNP, Luukkonen et al. introduced two different types of *PNPLA3* mutations into a human cell line using CRISPR/Cas9 genome editing [35]. They subsequently obtained a homozygous PNPLA3-knockout (KO) cell line containing a 2-bp deletion after 146C and a homozygous PNPLA3-I148M knock-in (KI) cell line with the specific C > G nucleotide substitution using an HDR donor. Both cell lines exhibited a frameshift within PNPLA3 and premature termination of translation. As expected, the PNPLA3-I148M-KI cell line exhibited increased levels of neutral lipids, including TGs. Introduction of unsaturated fatty acids into PNPLA3-KO and PNPLA3-I148M-KI mutant cells dramatically increased lipid droplet accumulation in both cell lines, an effect that was not observed upon introduction of saturated fatty acids. Collectively, these observations suggest that loss of PNPLA3 results in preferential sequestration of unsaturated fatty acids into neutral lipids. Thus, PNPLA3 may function as an unsaturated fatty acid-specific hydrolase.

#### 2.2.2. TM6SF2-Targeted Models

An earlier GWAS study revealed that the *TM6SF2* rs58542926 (C > T) variant is closely associated with elevated hepatic TG and serum AST levels [14,15,16]. To explore the pathophysiological role of *TM6SF2*, Fan et al. disrupted *TM6SF2* in mice using CRISPR/Cas9 genome editing [36], obtaining *TM6SF2* mutant mice containing a C/G-bp insertion immediately after the start codon. *TM6SF2* mutant mice exhibited a slight increase in serum TG levels, but showed no significant differences in hepatic TG accumulation or serum ALT and AST levels compared with wild-type mice—phenotypic features that did not correspond to those observed in human carriers of the *TM6SF2* rs58542926 variant [14]. Instead, these mice showed decreased plasma levels of high-density lipoprotein (HDL), LDL, and total cholesterol, findings consistent with human genetic studies [36]. These data indicate that *TM6SF2* plays a critical role in cholesterol metabolism. Another group disrupted *TM6SF2* in zebrafish using CRISPR/Cas9 by co-injecting larvae with Cas9 mRNA and gRNA targeting either exon 3 or exon 4 of *TM6SF2* [37]. Both mutant lines exhibited increased hepatic lipid accumulation.

#### 2.2.3. Other NAFLD Models

CRISPR/Cas9 genome editing has also been used to investigate functional roles of NAFLD-associated *GCKR* and *MBOAT7* variants. Codner et al. established GCKR P446L mice by HDR-mediated CRISPR/Cas9 genome editing using long single-stranded DNA templates [38], although their phenotypic outcomes have not yet been reported. Meroni et al. generated three *MBOA7*-deleted human liver cell lines containing 31-, 101-, or 917-bp deletions using NHEJ-mediated CRISPR/Cas9 genome editing [39]. All three mutant cell lines displayed increased intracellular fat content. The mutant cell line with the largest deletion (∆917 bp) showed barely detectable expression of the *MBOA7* gene and exhibited the most dramatic increase in fat accumulation, particularly saturated and mono-unsaturated TGs. These results indicate that MBOA7 plays a critical role in hepatic fat accumulation.

## 3. Selection of a “Good-Fit” Genome-Editing Tool for Replicating NAFLD Generic Variants

**Figure 2:**
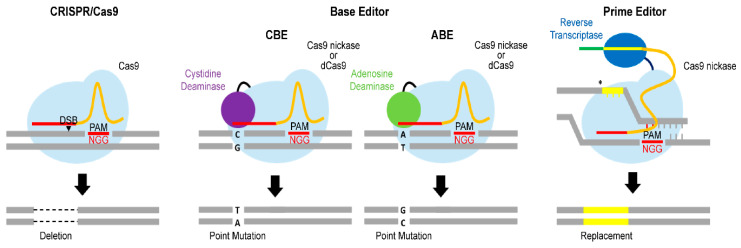
Schematic depiction of CRISPR/Cas9, base editors, and a prime editor. Three different types of genome-editing tools and their outcomes are shown. Left: CRISPR/Cas9 primarily generates double-strand break (DSB)-mediated deletions. Middle: Base editors generate point mutations using cytidine deaminase (C to T or T to C) or adenosine deaminase (A to G or G to A) without inducing DSBs. Right: A prime editor replaces specific sequences (<10 bp) using reverse transcriptase without the need for DSBs or donor DNA templates. dCas9, dead Cas9.

Although CRISPR/Cas9 has opened a new era in genome editing, it still has some limitations in generating target-specific point mutations or precise sequence replacements. Indeed, the efficiency of HDR-mediated nucleotide insertion is very low (<5%) [40]. NHEJ-mediated CRISPR/Cas9 genome editing can also generate point mutations or insertions, but these are random events [34]. To overcome these issues, researchers have attempted to generate advanced genome-editing tools using Cas9 variants [41]. Below, we describe representative Cas9 variants that can be useful for reassembling NAFLD SNPs (Figure 2). Selection of a good-fit genome-editing tool will aid the successful establishment of research models mirroring genuine genetic features of NAFLD.

### 3.1. Advanced Cas9 Variants

#### 3.1.1. Base Editor

A base editor for generating specific point mutations without the need for donor DNA templates was first developed by Liu and colleagues using a Cas9 variant [42]. The resulting base editor uses catalytically impaired Cas9 nucleases (e.g., deadCas9 or Cas9 nickase) that are incapable of inducing DSBs [43]. An engineered Cas9 was further linked to cytosine deaminase, an enzyme that can substitute cytosine (C) for thymine (T) or T for C. Although this cytosine base editor (CBE) is very efficient in generating point mutations, its ability to perform nucleotide substitutions is limited to C-to-T conversions and vice versa. To broaden the range of target nucleotides, Liu and co-workers further developed an adenosine base editor (ABE) by linking adenosine deaminase to dCas9 or Cas9 nickase [44]. ABE enables mutation of adenine (A) to guanine (G) or G to A. Since both CBE and ABE show relatively high targeting efficiency (>70%), they are good options for generating specific point mutations. Therefore, base editors would be useful for substituting a single nucleotide that copied NAFLD variants. In particular, CBE would be the appropriate option for mirroring *TM6SF2* rs58542926 (C > T), *TM6SF2* rs10401969 (C > T), *GCKR* rs1260326 (T > C), *GCKR* rs780094 (C > T), and *MBOAT7* (rs641738, C > T) variants.

#### 3.1.2. Prime Editor

Although base editors allow a specific single nucleotide to be mutated, replacement of a short stretch of DNA is still technically difficult. Liu and co-workers further developed a prime editor that enables insertions and point mutations without the need for DSBs and donor DNA templates [45]. The unique feature of the prime editor is its use of a prime editing gRNA (pegRNA) and a Cas9 nickase linked to reverse transcriptase. pegRNA is an extended gRNA containing a primer binding site and reverse transcriptase template sequence. Once pegRNA recognizes the target sequence, Cas9 nickase introduces a nick adjacent to the PAM. Reverse transcriptase further synthesizes a new DNA fragment using the sequence at the 3′-end of pegRNA as a template. The newly synthesized DNA strand is incorporated into the nicked DNA strand and then the complementary strand is repaired using the genome-edited strand as a template. Consequently, the prime editor can generate all types of mutations, including sequence replacements, point mutations, short deletions, and short insertions. The prime editor has several advantages over CRISPR/Cas or base editors. It can generate more precise insertions or point mutations than NHEJ-mediated CRISPR/Cas9 genome editing, and because the prime editor is capable of incorporating a point mutation far from the nick site (>30 bp), its target range is more flexible than that of HDR-mediated insertion [41]. Unlike base editors, the prime editor can generate both transition (purine to purine; C↔T or G↔A) and transversion (purine to pyrimidine; A or G to T or C) mutations [45,46]. These benefits enable a prime editor to create NAFLD polymorphisms that base editors cannot generate, such as *PNPLA3* rs738409 (C > G) and *HMOX1* rs2071746 (A > T).

### 3.2. Various Gene-Delivery Methods for Optimizing Genome-Editing Efficiency

Recent advances in genome-editing tools have been accompanied by investigations of various gene delivery methods for improving gene-editing efficiency (Table 3). gRNA and Cas9 can be introduced into cells in three different platforms: (1) gRNA and Cas9 mRNA, (2) DNA plasmids encoding gRNA and/or Cas9, and (3) gRNA and Cas9 protein. In earlier studies, Cas9 was frequently introduced into cells and mouse embryos in the form of mRNA or DNA plasmids [31,47,48,49]. Later, Cas9 was introduced in the form of a ribonucleoprotein, reflecting several reports that the ribonucleoprotein induces a lower off-target cleavage rate than plasmid DNA [50,51,52]. For delivery of CRISPR/Cas9 into cells, electroporation has been the most common in vitro method for cell lines [32] and microinjection has been the standard method for in vivo animal models [53,54,55]. Both delivery methods have been well characterized and guaranteed gene delivery. However, the efficiency of electroporation is largely dependent on cell type, and microinjection is technically challenging, requiring well-trained personnel [56,57]. Cas variants, such as base editors and prime editors, have been more frequently introduced in the form of plasmid DNA [42,44,45]. Plasmid DNA is primarily delivered using liposomes because they can be easily managed and are cost-effective, but they are limited with respect to in vivo delivery [56]. Endosomal degradation can affect liposome-mediated delivery efficiency, and some cell types are not amenable to this technique. Gold nanoparticles can be a good alternative to liposomes; they can efficiently deliver Cas9 in the form of mRNA or protein to both cells and organs [58], and they are inert, inducing little or no immune response. However, they need to be better characterized for clinical applications.

Various types of viral vectors for gene editing have been used to further improve the efficiency of gene delivery. One widely used viral vector is adeno-associated virus (AAV) [63], which can efficiently infect both dividing and non-dividing cells. However, their genome packaging capacity (~4.7 kb) is small relative to the size of Cas9 (4.1 kb), and the total size becomes larger if a donor DNA template needs to be introduced. This has led to the development of smaller Cas9 variants, such as Cpf (3.9 kb), saCas9 (3.16 kb), and cjCas9 (2.95 kb) [65,69,82,83]. Adenoviruses have a much larger genome capacity (~30 kb) than AAVs, but they can induce a strong adaptive immune response in host cells [56]. Lentiviral or retroviral vectors have a somewhat larger genome capacity (~7 kb) than AAVs, but these viral vectors are well known for their ability to induce unwanted viral genome integration into host cells [58]. They also elicit strong immune responses. Several more recent studies have used baculoviral vectors for genome editing [80,81]. Since baculoviral vectors have a large genome capacity (>100 kb), the size of Cas9 or Cas9 variants is not a concern [84]. Baculoviruses are safer than retroviruses because, although they can infect mammalian cells, they can only replicate in their original host insect cells [85]. They also have a low propensity to induce immune responses, but further studies are needed for clinical applications.

## 4. Discussion

NAFLD, a multifactorial disease influenced by both environmental and genetic factors, is an increasingly common liver disorder worldwide, especially in developing countries [6]. Although NAFLD is greatly affected by environmental factors such as dietary pattern and sedentary lifestyle, genetic factors provide the basis for disease onset and severity. Recent GWAS and cohort studies have identified several SNPs associated with NAFLD [11,14,17,20,26]. However, without functional studies, their pathophysiological roles in NAFLD progression cannot be truly verified. Prior to adoption of the CRISPR/Cas9 genome-editing technique, NAFLD models were generated using siRNA, shRNA, morpholinos, or conventional Cre/loxP recombination [14,86,87,88]. However, these tools are incapable of copying NAFLD-associated SNPs because, despite being able to inhibit specific gene expression, they are incapable of generating single-nucleotide substitutions. Although the Cre/loxP recombination system enables mutation to a specific nucleotide, it is technically challenging and laborious. Since its development, the CRISPR/Cas9 genome-editing tool has been rapidly adopted for generation of NAFLD models [35,36,38,39]. However, NHEJ-mediated CRISPR/Cas9 primarily produces gene deletions, and the efficiency of HDR-mediated insertion is quite low. Accordingly, some results obtained from NAFLD models are not well correlated with human data. Hence, we suggest using advanced Cas9 variants, such as base editors and a primer editor, to introduce SNPs to generate NAFLD models. CBE and ABE would be suitable for generating purine-to-purine mutations, whereas a prime editor would be more appropriate for establishing purine-to-pyrimidine mutations. These tools will allow more precise and sophisticated genome editing.

## 5. Future Directions

CRISPR/Cas9 and various Cas9 variants have revolutionized the genome-editing field; however, unwanted off-target events have been problematic from both basic research and clinical application standpoints [89,90]. It is well known that CRISPR/Cas9 generates frequent off-target events. Base editors can also generate some unwanted bystander edits [41], but recent whole-genome sequencing analyses have shown that ABEs induce much lower off-target effects than CBEs [91]. Further studies are needed to characterize the off-target activity of prime editors. Selection of an appropriate gene delivery method is crucial for further optimizing the efficiency of genome editing. Currently available gene-delivery methods have both benefits and shortcomings. Although more studies are needed, gold nanoparticles and baculoviral vectors would be good options for in vitro and in vivo delivery of genome-editing tools, respectively. A systematic strategy for selecting a good-fit genome-editing tool and appropriate gene-delivery method should promote the successful establishment of genuine NAFLD models. Such NAFLD models will ultimately contribute to identifying the molecular pathophysiology underlying NAFLD onset and progression. Moreover, NAFLD SNPs can be used as biomarkers for genomic diagnosis and as a clinical target for gene therapy.

## 6. Conclusions

Recent progress in genome engineering has revolutionized the establishment of various disease models that mimic genetic variants. In vitro and in vivo models of NAFLD have also been generated using CRISPR/Cas9 genome editing. In addition to CRISPR/Cas9, which primarily generates deletion mutations, Cas9 variants, such as base editors and prime editors, are available for specifically generating point mutations or sequence replacements. Selection of the most suitable genome-editing tool together with an efficient gene delivery method will lead to the successful establishment of NAFLD models for the development of therapeutics.

## Figures and Tables

**Table 1:** Representative genetic variations associated with NAFLD.

Gene	Function	Variant	Phenotype of Genetic Variant	Reference
*PNPLA3*	TG lipase	rs738409 C > G (I148M)	Increased hepatic fat content, high risk of hepatic steatosis, fibrosis, and HCC	[11,12,13]
*TM6SF2*	Involved in very low-density lipoprotein (VLDL) secretion and hepatic TG metabolism	rs58542926 C > T (E167K)	Elevated hepatic TG, serum ALT, and AST levels	[14,15]
		rs10401969 C > T (intron)	Low hepatic TM6SF2 mRNA levels correlated with larger hepatocellular lipid droplets	[16]
*GCKR*	Regulator of glucokinase	rs1260326 T>C/T>G (P446L)	Increased hepatic TG and LDL-cholesterol levels, correlated with the severity of NASH	[17]
		rs780094 C > T	Increased hepatic TG levels, high risk of liver fibrosis	[18,19]
*MBOAT7*	Reacylation of phospholipid	rs641738 C > T	Increased hepatic TG levels and severity of NAFLD, high risk of HCC	[20,21,22,23]
*HMOX1*	Protects the liver against oxidative stress	rs2071746 A > T (promoter)	Increased serum ALT levels in pediatric NAFLD patients	[24,25,26]

**Table 2:** Clustered regularly interspaced short palindromic repeats/CRISPR-associated protein 9 (CRISPR/Cas9)-mediated NAFLD models.

Target Gene	Model Organism	Genotypic Outcomes	Delivery of Cas9	Phenotypic Effects	Ref.
*PNPLA3* (rs738409)	Human epidermal carcinoma cell line (A431)	2-bp deletion after 146C	Transfection	Increased lipid droplet accumulation	[35]
		I148M	Transfection	Increased levels of neutral lipids	[35]
*TM6SF2* (rs58542926)	Mouse	C/G insertion after start codon	Microinjection	Increased serum TG levels with a high-fat diet	[36]
	Zebrafish	Partial deletion of exon 3 or exon 4	Microinjection	Increased hepatic lipid accumulation	[37]
*GCKR* (rs1260326)	Mouse	P446L	Microinjection	N/A	[38]
*MBOAT7* (rs641738)	Human HCC cell line (HepG2)	31-bp, 91-bp, or 101-bp deletion	N/A	Increased fat accumulation	[39]

**Table 3:** Various gene-delivery methods for genome editing.

Type	Delivery Methods	Advantages	Limitations	Reference
Non-viral	Electroporation/nucleofection	Delivery to cell population, well-characterized	In vitro only; some cells are not suitable	[32,51]
	Microinjection	Efficient germ line delivery	Technically challenging, laborious	[48,49,59,60]
	Liposomes	Simple, easy to manipulate, low cost	In vitro only, endosomal degradation of cargo, specific cell tropism	[31,42,44,45]
	Gold nanoparticles (AuNPs)	Inert, low immune responses	Not well characterized	[52,61]
Viral	Adeno-associated virus (AAV)	Efficient delivery to both dividing and non-dividing cells	Very low genome capacity (<5 kb nucleic acid)	[62,63,64,65,66,67,68,69]
	Adenovirus	High delivery efficiency, high genome capacity (up to 30 kb nucleic acid)	Adaptive immune responses	[70,71,72,73]
	Retrovirus	High delivery efficiency, persistent gene expression	Only dividing cells can be infected, unwanted viral genome integration	[74,75,76]
	Lentivirus	Long-term gene expression, relatively high genome capacity, (~10 kb)	Unwanted viral genome integration, strong immune responses	[77,78,79]
	Baculovirus	Very high genome capacity (>100 kb), minimal immunogenicity	Not well characterized	[80,81]

## References

[1] Brunt E.M., Wong V.W., Nobili V., Day C.P., Sookoian S., Maher J.J., Bugianesi E., Sirlin C.B., Neuschwander-Tetri B.A., Rinella M.E. (2015). Nonalcoholic fatty liver disease. Nat. Rev. Dis. Primers.

[2] Anstee Q.M., Day C.P. (2013). The genetics of NAFLD. Nat. Rev. Gastroenterol. Hepatol..

[3] Bettermann K., Hohensee T., Haybaeck J. (2014). Steatosis and steatohepatitis: Complex disorders. Int. J. Mol. Sci..

[4] Haybaeck J., Stumptner C., Thueringer A., Kolbe T., Magin T.M., Hesse M., Fickert P., Tsybrovskyy O., Muller H., Trauner M. (2012). Genetic background effects of keratin 8 and 18 in a DDC-induced hepatotoxicity and Mallory-Denk body formation mouse model. Lab. Investig..

[5] Zatloukal B., Kufferath I., Thueringer A., Landegren U., Zatloukal K., Haybaeck J. (2014). Sensitivity and specificity of in situ proximity ligation for protein interaction analysis in a model of steatohepatitis with Mallory-Denk bodies. PLoS ONE.

[6] Younossi Z.M., Koenig A.B., Abdelatif D., Fazel Y., Henry L., Wymer M. (2016). Global epidemiology of nonalcoholic fatty liver disease-Meta-analytic assessment of prevalence, incidence, and outcomes. Hepatology.

[7] Bettermann K., Mehta A.K., Hofer E.M., Wohlrab C., Golob-Schwarzl N., Svendova V., Schimek M.G., Stumptner C., Thuringer A., Speicher M.R. (2016). Keratin 18-deficiency results in steatohepatitis and liver tumors in old mice: A model of steatohepatitis-associated liver carcinogenesis. Oncotarget.

[8] Golob-Schwarzl N., Bettermann K., Mehta A.K., Kessler S.M., Unterluggauer J., Krassnig S., Kojima K., Chen X., Hoshida Y., Bardeesy N.M. (2019). High Keratin 8/18 Ratio Predicts Aggressive Hepatocellular Cancer Phenotype. Transl. Oncol..

[9] Severson T.J., Besur S., Bonkovsky H.L. (2016). Genetic factors that affect nonalcoholic fatty liver disease: A systematic clinical review. World J. Gastroenterol..

[10] Machado M.V., Cortez-Pinto H. (2014). Non-alcoholic fatty liver disease: What the clinician needs to know. World J. Gastroenterol..

[11] Romeo S., Kozlitina J., Xing C., Pertsemlidis A., Cox D., Pennacchio L.A., Boerwinkle E., Cohen J.C., Hobbs H.H. (2008). Genetic variation in PNPLA3 confers susceptibility to nonalcoholic fatty liver disease. Nat. Genet..

[12] Sookoian S., Castano G.O., Burgueno A.L., Gianotti T.F., Rosselli M.S., Pirola C.J. (2009). A nonsynonymous gene variant in the adiponutrin gene is associated with nonalcoholic fatty liver disease severity. J. Lipid Res..

[13] Liu Y.L., Patman G.L., Leathart J.B., Piguet A.C., Burt A.D., Dufour J.F., Day C.P., Daly A.K., Reeves H.L., Anstee Q.M. (2014). Carriage of the PNPLA3 rs738409 C > G polymorphism confers an increased risk of non-alcoholic fatty liver disease associated hepatocellular carcinoma. J. Hepatol..

[14] Kozlitina J., Smagris E., Stender S., Nordestgaard B.G., Zhou H.H., Tybjaerg-Hansen A., Vogt T.F., Hobbs H.H., Cohen J.C. (2014). Exome-wide association study identifies a TM6SF2 variant that confers susceptibility to nonalcoholic fatty liver disease. Nat. Genet..

[15] Liu Y.L., Reeves H.L., Burt A.D., Tiniakos D., McPherson S., Leathart J.B., Allison M.E., Alexander G.J., Piguet A.C., Anty R. (2014). TM6SF2 rs58542926 influences hepatic fibrosis progression in patients with non-alcoholic fatty liver disease. Nat. Commun..

[16] Mahdessian H., Taxiarchis A., Popov S., Silveira A., Franco-Cereceda A., Hamsten A., Eriksson P., van’t Hooft F. (2014). TM6SF2 is a regulator of liver fat metabolism influencing triglyceride secretion and hepatic lipid droplet content. Proc. Natl. Acad. Sci. USA.

[17] Beer N.L., Tribble N.D., McCulloch L.J., Roos C., Johnson P.R., Orho-Melander M., Gloyn A.L. (2009). The P446L variant in GCKR associated with fasting plasma glucose and triglyceride levels exerts its effect through increased glucokinase activity in liver. Hum. Mol. Genet..

[18] Speliotes E.K., Yerges-Armstrong L.M., Wu J., Hernaez R., Kim L.J., Palmer C.D., Gudnason V., Eiriksdottir G., Garcia M.E., Launer L.J. (2011). Genome-wide association analysis identifies variants associated with nonalcoholic fatty liver disease that have distinct effects on metabolic traits. PLoS Genet..

[19] Petta S., Miele L., Bugianesi E., Camma C., Rosso C., Boccia S., Cabibi D., Di Marco V., Grimaudo S., Grieco A. (2014). Glucokinase regulatory protein gene polymorphism affects liver fibrosis in non-alcoholic fatty liver disease. PLoS ONE.

[20] Buch S., Stickel F., Trepo E., Way M., Herrmann A., Nischalke H.D., Brosch M., Rosendahl J., Berg T., Ridinger M. (2015). A genome-wide association study confirms PNPLA3 and identifies TM6SF2 and MBOAT7 as risk loci for alcohol-related cirrhosis. Nat. Genet..

[21] Mancina R.M., Dongiovanni P., Petta S., Pingitore P., Meroni M., Rametta R., Boren J., Montalcini T., Pujia A., Wiklund O. (2016). The MBOAT7-TMC4 Variant rs641738 Increases Risk of Nonalcoholic Fatty Liver Disease in Individuals of European Descent. Gastroenterology.

[22] Viitasalo A., Eloranta A.M., Atalay M., Romeo S., Pihlajamaki J., Lakka T.A. (2016). Association of MBOAT7 gene variant with plasma ALT levels in children: The PANIC study. Pediatr. Res..

[23] Donati B., Dongiovanni P., Romeo S., Meroni M., McCain M., Miele L., Petta S., Maier S., Rosso C., De Luca L. (2017). MBOAT7 rs641738 variant and hepatocellular carcinoma in non-cirrhotic individuals. Sci. Rep..

[24] Salley T.N., Mishra M., Tiwari S., Jadhav A., Ndisang J.F. (2013). The heme oxygenase system rescues hepatic deterioration in the condition of obesity co-morbid with type-2 diabetes. PLoS ONE.

[25] Hinds T.D., Sodhi K., Meadows C., Fedorova L., Puri N., Kim D.H., Peterson S.J., Shapiro J., Abraham N.G., Kappas A. (2014). Increased HO-1 levels ameliorate fatty liver development through a reduction of heme and recruitment of FGF21. Obesity.

[26] Chang P.F., Lin Y.C., Liu K., Yeh S.J., Ni Y.H. (2015). Heme oxygenase-1 gene promoter polymorphism and the risk of pediatric nonalcoholic fatty liver disease. Int. J. Obes..

[27] Gaj T., Gersbach C.A., Barbas C.F. (2013). ZFN, TALEN, and CRISPR/Cas-based methods for genome engineering. Trends Biotechnol..

[28] Alves-Bezerra M., Furey N., Johnson C.G., Bissig K.D. (2019). Using CRISPR/Cas9 to model human liver disease. JHEP Rep..

[29] Barrangou R. (2015). The roles of CRISPR-Cas systems in adaptive immunity and beyond. Curr. Opin. Immunol..

[30] Jinek M., Chylinski K., Fonfara I., Hauer M., Doudna J.A., Charpentier E. (2012). A programmable dual-RNA-guided DNA endonuclease in adaptive bacterial immunity. Science.

[31] Cong L., Ran F.A., Cox D., Lin S., Barretto R., Habib N., Hsu P.D., Wu X., Jiang W., Marraffini L.A. (2013). Multiplex genome engineering using CRISPR/Cas systems. Science.

[32] Mali P., Yang L., Esvelt K.M., Aach J., Guell M., DiCarlo J.E., Norville J.E., Church G.M. (2013). RNA-guided human genome engineering via Cas9. Science.

[33] Hsu P.D., Lander E.S., Zhang F. (2014). Development and applications of CRISPR-Cas9 for genome engineering. Cell.

[34] Shin H.Y., Wang C., Lee H.K., Yoo K.H., Zeng X., Kuhns T., Yang C.M., Mohr T., Liu C., Hennighausen L. (2017). CRISPR/Cas9 targeting events cause complex deletions and insertions at 17 sites in the mouse genome. Nat. Commun..

[35] Luukkonen P.K., Nick A., Holtta-Vuori M., Thiele C., Isokuortti E., Lallukka-Bruck S., Zhou Y., Hakkarainen A., Lundbom N., Peltonen M. (2019). Human PNPLA3-I148M variant increases hepatic retention of polyunsaturated fatty acids. JCI Insight.

[36] Fan Y., Lu H., Guo Y., Zhu T., Garcia-Barrio M.T., Jiang Z., Willer C.J., Zhang J., Chen Y.E. (2016). Hepatic Transmembrane 6 Superfamily Member 2 Regulates Cholesterol Metabolism in Mice. Gastroenterology.

[37] O’Hare E.A., Yang R., Yerges-Armstrong L.M., Sreenivasan U., McFarland R., Leitch C.C., Wilson M.H., Narina S., Gorden A., Ryan K.A. (2017). TM6SF2 rs58542926 impacts lipid processing in liver and small intestine. Hepatology.

[38] Codner G.F., Mianne J., Caulder A., Loeffler J., Fell R., King R., Allan A.J., Mackenzie M., Pike F.J., McCabe C.V. (2018). Application of long single-stranded DNA donors in genome editing: Generation and validation of mouse mutants. BMC Biol..

[39] Meroni M., Dongiovanni P., Longo M., Carli F., Baselli G., Rametta R., Pelusi S., Badiali S., Maggioni M., Gaggini M. (2020). Mboat7 down-regulation by hyper-insulinemia induces fat accumulation in hepatocytes. EBioMedicine.

[40] Liu M., Rehman S., Tang X., Gu K., Fan Q., Chen D., Ma W. (2018). Methodologies for Improving HDR Efficiency. Front. Genet..

[41] Anzalone A.V., Koblan L.W., Liu D.R. (2020). Genome editing with CRISPR-Cas nucleases, base editors, transposases and prime editors. Nat. Biotechnol..

[42] Komor A.C., Kim Y.B., Packer M.S., Zuris J.A., Liu D.R. (2016). Programmable editing of a target base in genomic DNA without double-stranded DNA cleavage. Nature.

[43] Ran F.A., Hsu P.D., Lin C.Y., Gootenberg J.S., Konermann S., Trevino A.E., Scott D.A., Inoue A., Matoba S., Zhang Y. (2013). Double nicking by RNA-guided CRISPR Cas9 for enhanced genome editing specificity. Cell.

[44] Gaudelli N.M., Komor A.C., Rees H.A., Packer M.S., Badran A.H., Bryson D.I., Liu D.R. (2017). Programmable base editing of A*T to G*C in genomic DNA without DNA cleavage. Nature.

[45] Anzalone A.V., Randolph P.B., Davis J.R., Sousa A.A., Koblan L.W., Levy J.M., Chen P.J., Wilson C., Newby G.A., Raguram A. (2019). Search-and-replace genome editing without double-strand breaks or donor DNA. Nature.

[46] Paquet D., Kwart D., Chen A., Sproul A., Jacob S., Teo S., Olsen K.M., Gregg A., Noggle S., Tessier-Lavigne M. (2016). Efficient introduction of specific homozygous and heterozygous mutations using CRISPR/Cas9. Nature.

[47] Chen S., Lee B., Lee A.Y., Modzelewski A.J., He L. (2016). Highly Efficient Mouse Genome Editing by CRISPR Ribonucleoprotein Electroporation of Zygotes. J. Biol. Chem..

[48] Shin H.Y., Willi M., HyunYoo K., Zeng X., Wang C., Metser G., Hennighausen L. (2016). Hierarchy within the mammary STAT5-driven Wap super-enhancer. Nat. Genet..

[49] Yang H., Wang H., Shivalila C.S., Cheng A.W., Shi L., Jaenisch R. (2013). One-step generation of mice carrying reporter and conditional alleles by CRISPR/Cas-mediated genome engineering. Cell.

[50] Kim S., Kim D., Cho S.W., Kim J., Kim J.S. (2014). Highly efficient RNA-guided genome editing in human cells via delivery of purified Cas9 ribonucleoproteins. Genome Res..

[51] Liang X., Potter J., Kumar S., Zou Y., Quintanilla R., Sridharan M., Carte J., Chen W., Roark N., Ranganathan S. (2015). Rapid and highly efficient mammalian cell engineering via Cas9 protein transfection. J. Biotechnol..

[52] Mout R., Ray M., Yesilbag Tonga G., Lee Y.W., Tay T., Sasaki K., Rotello V.M. (2017). Direct Cytosolic Delivery of CRISPR/Cas9-Ribonucleoprotein for Efficient Gene Editing. ACS Nano.

[53] Horii T., Arai Y., Yamazaki M., Morita S., Kimura M., Itoh M., Abe Y., Hatada I. (2014). Validation of microinjection methods for generating knockout mice by CRISPR/Cas-mediated genome engineering. Sci. Rep..

[54] Chuang C.K., Chen C.H., Huang C.L., Su Y.H., Peng S.H., Lin T.Y., Tai H.C., Yang T.S., Tu C.F. (2017). Generation of GGTA1 Mutant Pigs by Direct Pronuclear Microinjection of CRISPR/Cas9 Plasmid Vectors. Anim. Biotechnol..

[55] Crispo M., Mulet A.P., Tesson L., Barrera N., Cuadro F., dos Santos-Neto P.C., Nguyen T.H., Creneguy A., Brusselle L., Anegon I. (2015). Efficient Generation of Myostatin Knock-Out Sheep Using CRISPR/Cas9 Technology and Microinjection into Zygotes. PLoS ONE.

[56] Lino C.A., Harper J.C., Carney J.P., Timlin J.A. (2018). Delivering CRISPR: A review of the challenges and approaches. Drug Deliv..

[57] Li L., Hu S., Chen X. (2018). Non-viral delivery systems for CRISPR/Cas9-based genome editing: Challenges and opportunities. Biomaterials.

[58] Biagioni A., Laurenzana A., Margheri F., Chilla A., Fibbi G., Del Rosso M. (2018). Delivery systems of CRISPR/Cas9-based cancer gene therapy. J. Biol. Eng..

[59] Raveux A., Vandormael-Pournin S., Cohen-Tannoudji M. (2017). Optimization of the production of knock-in alleles by CRISPR/Cas9 microinjection into the mouse zygote. Sci. Rep..

[60] Metser G., Shin H.Y., Wang C., Yoo K.H., Oh S., Villarino A.V., O’Shea J.J., Kang K., Hennighausen L. (2016). An autoregulatory enhancer controls mammary-specific STAT5 functions. Nucleic Acids Res..

[61] Lee K., Conboy M., Park H.M., Jiang F., Kim H.J., Dewitt M.A., Mackley V.A., Chang K., Rao A., Skinner C. (2017). Nanoparticle delivery of Cas9 ribonucleoprotein and donor DNA in vivo induces homology-directed DNA repair. Nat. Biomed. Eng..

[62] Hung S.S., Chrysostomou V., Li F., Lim J.K., Wang J.H., Powell J.E., Tu L., Daniszewski M., Lo C., Wong R.C. (2016). AAV-Mediated CRISPR/Cas Gene Editing of Retinal Cells In Vivo. Investig. Ophthalmol. Vis. Sci..

[63] Lau C.H., Suh Y. (2017). In vivo genome editing in animals using AAV-CRISPR system: Applications to translational research of human disease. F1000Res.

[64] Platt R.J., Chen S., Zhou Y., Yim M.J., Swiech L., Kempton H.R., Dahlman J.E., Parnas O., Eisenhaure T.M., Jovanovic M. (2014). CRISPR-Cas9 knockin mice for genome editing and cancer modeling. Cell.

[65] Ran F.A., Cong L., Yan W.X., Scott D.A., Gootenberg J.S., Kriz A.J., Zetsche B., Shalem O., Wu X., Makarova K.S. (2015). In vivo genome editing using Staphylococcus aureus Cas9. Nature.

[66] Swiech L., Heidenreich M., Banerjee A., Habib N., Li Y., Trombetta J., Sur M., Zhang F. (2015). In vivo interrogation of gene function in the mammalian brain using CRISPR-Cas9. Nat Biotechnol.

[67] Long C., Amoasii L., Mireault A.A., McAnally J.R., Li H., Sanchez-Ortiz E., Bhattacharyya S., Shelton J.M., Bassel-Duby R., Olson E.N. (2016). Postnatal genome editing partially restores dystrophin expression in a mouse model of muscular dystrophy. Science.

[68] Carroll K.J., Makarewich C.A., McAnally J., Anderson D.M., Zentilin L., Liu N., Giacca M., Bassel-Duby R., Olson E.N. (2016). A mouse model for adult cardiac-specific gene deletion with CRISPR/Cas9. Proc. Natl. Acad. Sci. USA.

[69] Kim E., Koo T., Park S.W., Kim D., Kim K., Cho H.Y., Song D.W., Lee K.J., Jung M.H., Kim S. (2017). In vivo genome editing with a small Cas9 orthologue derived from Campylobacter jejuni. Nat. Commun..

[70] Voets O., Tielen F., Elstak E., Benschop J., Grimbergen M., Stallen J., Janssen R., van Marle A., Essrich C. (2017). Highly efficient gene inactivation by adenoviral CRISPR/Cas9 in human primary cells. PLoS ONE.

[71] Maddalo D., Manchado E., Concepcion C.P., Bonetti C., Vidigal J.A., Han Y.C., Ogrodowski P., Crippa A., Rekhtman N., de Stanchina E. (2014). In vivo engineering of oncogenic chromosomal rearrangements with the CRISPR/Cas9 system. Nature.

[72] Wang D., Mou H., Li S., Li Y., Hough S., Tran K., Li J., Yin H., Anderson D.G., Sontheimer E.J. (2015). Adenovirus-Mediated Somatic Genome Editing of Pten by CRISPR/Cas9 in Mouse Liver in Spite of Cas9-Specific Immune Responses. Hum. Gene. Ther..

[73] Ding Q., Strong A., Patel K.M., Ng S.L., Gosis B.S., Regan S.N., Cowan C.A., Rader D.J., Musunuru K. (2014). Permanent alteration of PCSK9 with in vivo CRISPR-Cas9 genome editing. Circ. Res..

[74] Lindel F., Dodt C.R., Weidner N., Noll M., Bergemann F., Behrendt R., Fischer S., Dietrich J., Cartellieri M., Hamann M.V. (2019). TraFo-CRISPR: Enhanced Genome Engineering by Transient Foamy Virus Vector-Mediated Delivery of CRISPR/Cas9 Components. Mol. Ther. Nucleic Acids.

[75] Stenger D., Stief T.A., Kaeuferle T., Willier S., Rataj F., Schober K., Vick B., Lotfi R., Wagner B., Grunewald T.G.P. (2020). Endogenous TCR promotes in vivo persistence of CD19-CAR-T cells compared to a CRISPR/Cas9-mediated TCR knockout CAR. Blood.

[76] Vila-Navarro E., Fernandez-Castaner E., Rovira-Rigau M., Raimondi G., Vila-Casadesus M., Lozano J.J., Soubeyran P., Iovanna J., Castells A., Fillat C. (2020). MiR-93 is related to poor prognosis in pancreatic cancer and promotes tumor progression by targeting microtubule dynamics. Oncogenesis.

[77] Huo W., Zhao G., Yin J., Ouyang X., Wang Y., Yang C., Wang B., Dong P., Wang Z., Watari H. (2017). Lentiviral CRISPR/Cas9 vector mediated miR-21 gene editing inhibits the epithelial to mesenchymal transition in ovarian cancer cells. J. Cancer.

[78] Kabadi A.M., Ousterout D.G., Hilton I.B., Gersbach C.A. (2014). Multiplex CRISPR/Cas9-based genome engineering from a single lentiviral vector. Nucleic Acids Res..

[79] Annunziato S., Kas S.M., Nethe M., Yucel H., Del Bravo J., Pritchard C., Bin Ali R., van Gerwen B., Siteur B., Drenth A.P. (2016). Modeling invasive lobular breast carcinoma by CRISPR/Cas9-mediated somatic genome editing of the mammary gland. Genes Dev..

[80] Hindriksen S., Bramer A.J., Truong M.A., Vromans M.J.M., Post J.B., Verlaan-Klink I., Snippert H.J., Lens S.M.A., Hadders M.A. (2017). Baculoviral delivery of CRISPR/Cas9 facilitates efficient genome editing in human cells. PLoS ONE.

[81] Mansouri M., Ehsaei Z., Taylor V., Berger P. (2017). Baculovirus-based genome editing in primary cells. Plasmid.

[82] Yoo K.H., Hennighausen L., Shin H.Y. (2019). Dissecting Tissue-Specific Super-Enhancers by Integrating Genome-Wide Analyses and CRISPR/Cas9 Genome Editing. J. Mammary Gland Biol. Neoplasia.

[83] Kim H.K., Song M., Lee J., Menon A.V., Jung S., Kang Y.M., Choi J.W., Woo E., Koh H.C., Nam J.W. (2017). In vivo high-throughput profiling of CRISPR-Cpf1 activity. Nat. Methods.

[84] Ono C., Okamoto T., Abe T., Matsuura Y. (2018). Baculovirus as a Tool for Gene Delivery and Gene Therapy. Viruses.

[85] Volkman L.E., Goldsmith P.A. (1983). In Vitro Survey of Autographa californica Nuclear Polyhedrosis Virus Interaction with Nontarget Vertebrate Host Cells. Appl. Environ. Microbiol..

[86] Smagris E., BasuRay S., Li J., Huang Y., Lai K.M., Gromada J., Cohen J.C., Hobbs H.H. (2015). Pnpla3I148M knockin mice accumulate PNPLA3 on lipid droplets and develop hepatic steatosis. Hepatology.

[87] O’Hare E.A., Yerges-Armstrong L.M., Perry J.A., Shuldiner A.R., Zaghloul N.A. (2016). Assignment of Functional Relevance to Genes at Type 2 Diabetes-Associated Loci Through Investigation of beta-Cell Mass Deficits. Mol. Endocrinol..

[88] Tanaka Y., Shimanaka Y., Caddeo A., Kubo T., Mao Y., Kubota T., Kubota N., Yamauchi T., Mancina R.M., Baselli G. (2020). LPIAT1/MBOAT7 depletion increases triglyceride synthesis fueled by high phosphatidylinositol turnover. Gut.

[89] Zhang X.H., Tee L.Y., Wang X.G., Huang Q.S., Yang S.H. (2015). Off-target Effects in CRISPR/Cas9-mediated Genome Engineering. Mol. Ther. Nucleic Acids.

[90] Wang Y., Wang M., Zheng T., Hou Y., Zhang P., Tang T., Wei J., Du Q. (2020). Specificity profiling of CRISPR system reveals greatly enhanced off-target gene editing. Sci. Rep..

[91] Lee H.K., Willi M., Miller S.M., Kim S., Liu C., Liu D.R., Hennighausen L. (2018). Targeting fidelity of adenine and cytosine base editors in mouse embryos. Nat. Commun..

